# The deubiquitinase OTUD3 stabilizes ACTN4 to drive growth and metastasis of hepatocellular carcinoma

**DOI:** 10.18632/aging.203293

**Published:** 2021-08-10

**Authors:** Peiyi Xie, Yanglin Chen, Hongfei Zhang, Guichao Zhou, Qing Chao, Jiangwen Wang, Yue Liu, Jiayu Fang, Jing Xie, Jing Zhen, Zhiyuan Wang, Liang Hao, Da Huang

**Affiliations:** 1 Department of General Surgery, Second Affiliated Hospital of Nanchang University, Nanchang, Jiangxi, China; 2 Second Abdominal Surgery Department, Affiliated Tumor Hospital of Nanchang University, Nanchang, Jiangxi, China; 3 School of Basic Medical Sciences, Medical College of Nanchang University, Nanchang, Jiangxi, China; 4 Department of Orthopedics, The Fourth Affiliated Hospital of Nanchang University, Nanchang, Jiangxi, China; 5 Second College of Clinical Medicine, Zunyi Medical University, Zhuhai, Guangdong, China; 6 Second College of Clinical Medicine, Nanchang University, Nanchang, Jiangxi, China; 7 Department of Imaging Center, Second Affiliated Hospital of Nanchang University, Nanchang, Jiangxi, China; 8 Department of Orthopedics, Second Affiliated Hospital of Nanchang University, Nanchang, Jiangxi, China; 9 Department of Thyroid Surgery, Second Affiliated Hospital of Nanchang University, Nanchang, Jiangxi, China

**Keywords:** OTUD3, ACTN4, HCC, growth, metastasis

## Abstract

OTU domain-containing protein 3 (OTUD3), a deubiquitinating enzyme, has been shown to participate in progression of multiple malignancies. The accurate function of OTUD3 in hepatocellular carcinoma (HCC) progression remains elusive. We found that OTUD3 was significantly overexpressed in HCC, and higher OTUD3 expression was correlated with larger tumor size, more distant metastasis, and worse TNM stage. A series of gain- and loss-of-function assays were also performed to examine the oncogenic function of OTUD3 in promoting HCC cell growth and metastasis *in vitro*. Using a xenograft mouse model, we showed that OTUD3 accelerated HCC progression *in vivo*. Furthermore, alpha-actinin 4 (ACTN4) was identified as a downstream target of OTUD3 through mass spectrometry analysis, and the ACTN4 protein level was significantly related to OTUD3 expression. Additionally, OTUD3 directly bound with ACTN4 and deubiquitinated ACTN4 to stabilize it. Finally, ACTN4 was found to be essential for OTUD3-mediated HCC proliferation and metastasis *in vitro* and *in vivo*. Collectively, our findings identify the oncogenic role of OTUD3 in HCC and suggest that OTUD3 can be considered as a pivotal prognostic biomarker and a potential therapeutic target.

## INTRODUCTION

Hepatocellular carcinoma (HCC) is the second lethal malignancies and is currently the sixth most common cancer worldwide [1]. HCC is a lethal cancer with limited therapeutic options, and the 5-year survival rate of HCC is only approximately 14%–18%; mortality is mostly due to the high probability of metastasis and rapid growth [2]. Therefore, understanding the molecular mechanisms of HCC progression will provide effective therapeutic strategies to reduce HCC mortality.

Posttranslational modifications of proteins can be reversed by peptidases such as deubiquitinase or deubiquitylating enzymes (DUBs), which mediate cleavage and removal of ubiquitin chains from substrate proteins [3]. Cysteine protease DUBs can be classified into four subfamilies according to their ubiquitin-protease domains: ovarian tumor (OTU), ubiquitin-specific protease (USP), ubiquitin C-terminal hydrolase (UCH) and Machado Joseph disease protease (MJD) [4]. Most OTU deubiquitinases act as important modulators of multiple cellular cascades. Study has shown that OTUD5 plays an important role in interferon signaling [5] and that OTUD1 regulates the DNA damage response [6]. Additionally, OTUD7B and OTULIN play important roles in the NF-kB signaling pathway [7, 8]. Though it belongs to the OTU family, the cellular function of OTU domain-containing protein 3 (OTUD3) is seldom reported. However, an increasing number of investigations have emphasized the role of OTUD3 in cancer. Zhang et al. reported the suppressive function of the OTUD3-PTEN axis in breast cancer, and OTUD3 was found to stabilize PTEN through deubiquitylation [9]. Subsequently, their further study showed the oncogenic function of OTUD3 in lung carcinoma and that OTUD3 drives lung cancer progression by stabilizing glucose-regulated protein 78-kDa (GRP78) [10]. Additionally, another study revealed that OTUD3 downregulation accelerates the growth and motility of colorectal cancer cells [11].

Here, we demonstrated that OTUD3 was dramatically upregulated in HCC, and the relationship between OTUD3 expression and the clinicopathological characteristics of HCC patients was investigated. We further investigated the oncogenic function of OTUD3 in facilitating HCC cell growth and metastasis through *in vitro* and *in vivo* experiments. Additionally, we found a positive correlation between OTUD3 and ACTN4 expression, and OTUD3 was found to be able to deubiquitinate and stabilize ACTN4 to increase its protein level in HCC cells. At last, we showed that ACTN4 was essential for OTUD3-drived HCC progression. Thus, our research strongly suggests that OTUD3 might be a novel target for HCC therapy.

## RESULTS

### OTUD3 is aberrantly upregulated in HCC tissues and is significantly correlated with the prognosis of HCC patients

**Figure 1:**
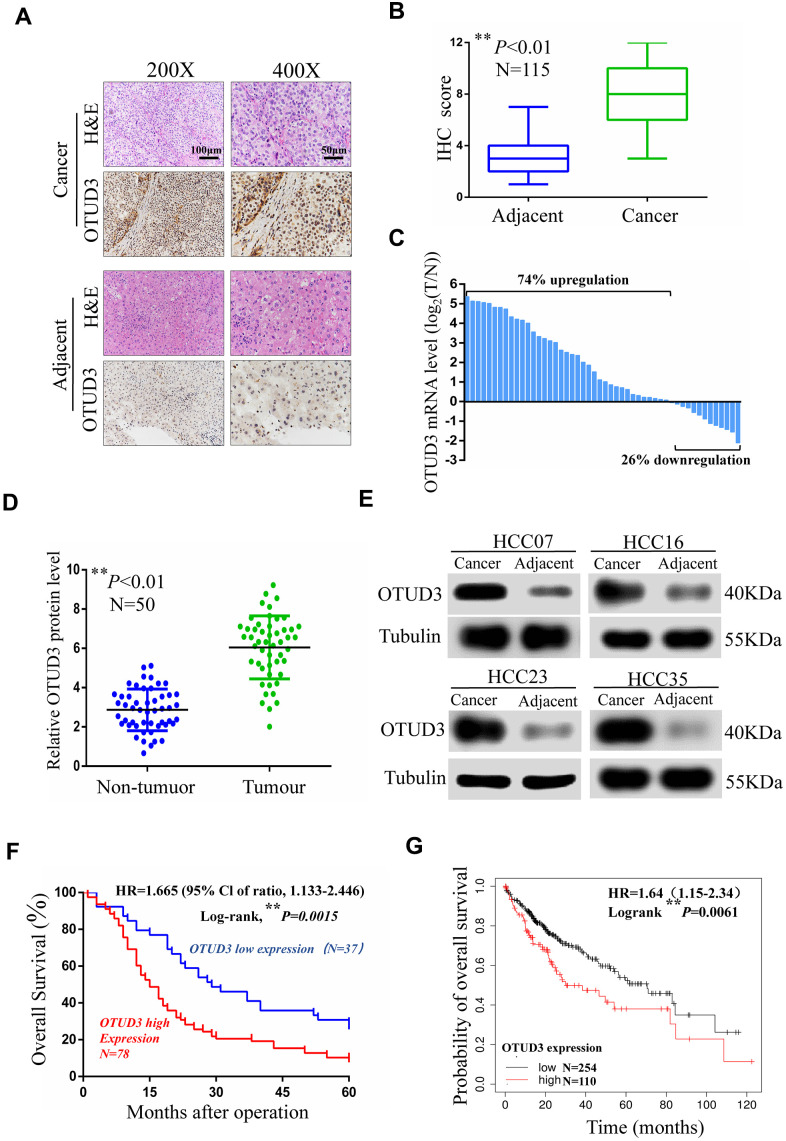
**OTUD3 is significantly upregulated in human HCC tissues.** (**A**) Representative images for OTUD3 IHC staining in HCC tissues and corresponding normal tissues (N=115; scale bar: 50μm, 100μm). (**B**) Diagram of OTUD3 staining score in IHC staining. (**C**) qRT-PCR analysis of OTUD3 mRNA level in 50 cases of HCC tissues and corresponding normal tissues. Left, a log 2 (T/N) value> 0 indicates that OTUD3 expression is overexpressed in the HCC samples; right, a log 2 (T/N) value< 0 indicates that OTUD3 expression is downregulated in the HCC samples. The OTUD3 mRNA levels are normalized to the GAPDH mRNA levels. (**D**) Determination and quantification of OTUD3 protein levels in HCC tissues and paired non-tumour tissues by western blot (N=50). (**E**) Representative images of western blot. (**F**) Kaplan–Meier analysis of the correlation between the OTUD3 level and overall survival of HCC patients with high and low OTUD3 expression in the IHC staining. (**G**) Kaplan–Meier analysis of OTUD3 expression in 364 liver cancer tissues using the Kaplan–Meier plotter website (https://www.kmplot.com). Data were mean ± S.D. of three independent determinations. Student’s t-test was used for P value assessment. *P< 0.05, **P<0.01.

To examine the expression level of OTUD3 in HCC tissues, we performed IHC staining and examined 115 pairs (including 50 pairs of fresh tissues) of paraffin-embedded archived HCC and paracancerous tissues. Our data indicated that OTUD3 was significantly overexpressed in HCC tissues (Figure 1A, 1B). Consistently, we employed qRT-PCR and western blot to detect 50 pairs of fresh HCC tissues. The qRT-PCR demonstrated that the mRNA expression of OTUD3 was dramatically upregulated in 37 of 50 HCC specimens in comparation with the level in adjacent normal tissues (Figure 1C). Western blot also indicated that the OTUD3 protein was markedly overexpressed in HCC tissues (Figure 1D, 1E). Furthermore, we examined the relationship of OTUD3 levels with different clinicopathological characteristics and found that higher OTUD3 expression was correlated with larger tumor size, more vascular invasion, intrahepatic metastasis and worse TNM stage (Table 1). Univariate survival analysis demonstrated that patients with high OTUD3 expression had worse overall survival than those with low OTUD3 expression (Figure 1F). Additionally, univariate and multivariate logistic regression analyses showed that OTUD3 was an independent predictor of poor prognosis for patients with HCC (Table 2). To further verify whether OTUD3 expression was correlated with a poor prognosis, we employed Kaplan–Meier analysis through the Kaplan–Meier plotter website. The results demonstrated that liver cancer patients with high OTUD3 expression had a markedly poorer overall survival probability than those with low OTUD3 expression. Taken together, these results show that OTUD3 was dramatically overexpressed in HCC tissues and that increased OTUD3 levels were correlated with advanced disease and poor patient prognosis.

**Table 1:** Relationship between OTUD3 expression and clinicopathological features in 115 HCC patients.

**Parameters**	**Total case**	**OTUD3**		**P value**
	**115**	**High expression**	**Low expression**	
Age(years)				*0.205*
<60	68	43	25	
≥60	47	35	12	
Gender				*0.242*
Female	43	32	11	
Male	72	46	26	
Tumor size (cm)				0.016*
<5	36	30	6	
≥5	79	48	31	
Tumor nodule number				*0.178*
Single	61	38	23	
Multiple	54	40	14	
AFP(ng/ml)				*0.363*
<400	52	33	19	
≧400	63	45	18	
Cirrhosis				*0.141*
Absence	36	21	15	
Presence	79	57	22	
Liver function				*0.448*
Child-Pugh A	32	20	12	
Child-Pugh B	83	58	25	
Lobe				*0.228*
Right	85	55	30	
Left	30	23	7	
TNM				0.034*
I/II	46	26	20	
III/IV	69	52	17	
Vascular invasion				0.042*
Negative	33	27	6	
Positive	82	51	31	
Intrahepatic metastasis				0.045*
Negative	41	23	18	
Positive	74	55	19	

Abbreviations: AFP, α-fetoprotein; TNM, tumor–node–metastasis. *P<0.05.

**Table 2:** Univariate and multivariate analysis of OTUD3 in overall survival of 115 HCC patients (Cox proportional hazards regression model).

**Parameters**	**Univariate analysis**			**Multivariate analysis**		
	**HR**	**95%CI**	**P value**	**HR**	**95%CI**	**P value**
Age (≥60/<60)	0.833	0.631-1.305	0.531	—	—	—
Gender (Male/Female)	1.875	1.079-2.759	0.177		—	—
Tumor nodule number (Single/Multiple)	1.164	0.631-2.153	0.467	—	—	—
AFP(ng/ml) (<400/≧400)	0.653	0.322-1.316	0.275	—	—	—
Cirrhosis (Absence/Presence)	0.756	0.431-1.623	0.335	—	—	—
Liver function (Child-Pugh A/Child-Pugh B)	1.632	0.731-3.115	0.312	—	—	—
Lobe (Right/Left)	0.693	0.413-1.531	0.364	—	—	—
Tumor size (≥5/<5)	1.862	0.952-2.879	<0.01**	1.503	0.911-2.651	0.035*
TNM (III, IV/I, II)	2.431	1.469-4.021	<0.01**	2.213	1.336-4.536	0.012*
Vascular invasion (Positive/Negative)	2.512	1.381-3.513	<0.01**	1.756	1.351-3.125	0.031*
Intrahepatic metastasis (Positive/Negative)	2.058	1.617-3.031	<0.01**	1.739	1.412-2.814	0.026*
OTUD3 expression (High/Low)	2.213	1.271-3.731	<0.01**	1.82	1.572-2.681	0.016*

Abbreviation: HR, hazard ratio; CI, confidence interval; AFP, α-fetoprotein; TNM, tumor–node–metastasis. *P<0.05.

### OTUD3 suppression inhibits HCC cell growth *in vitro* and *in vivo*

**Figure 2:**
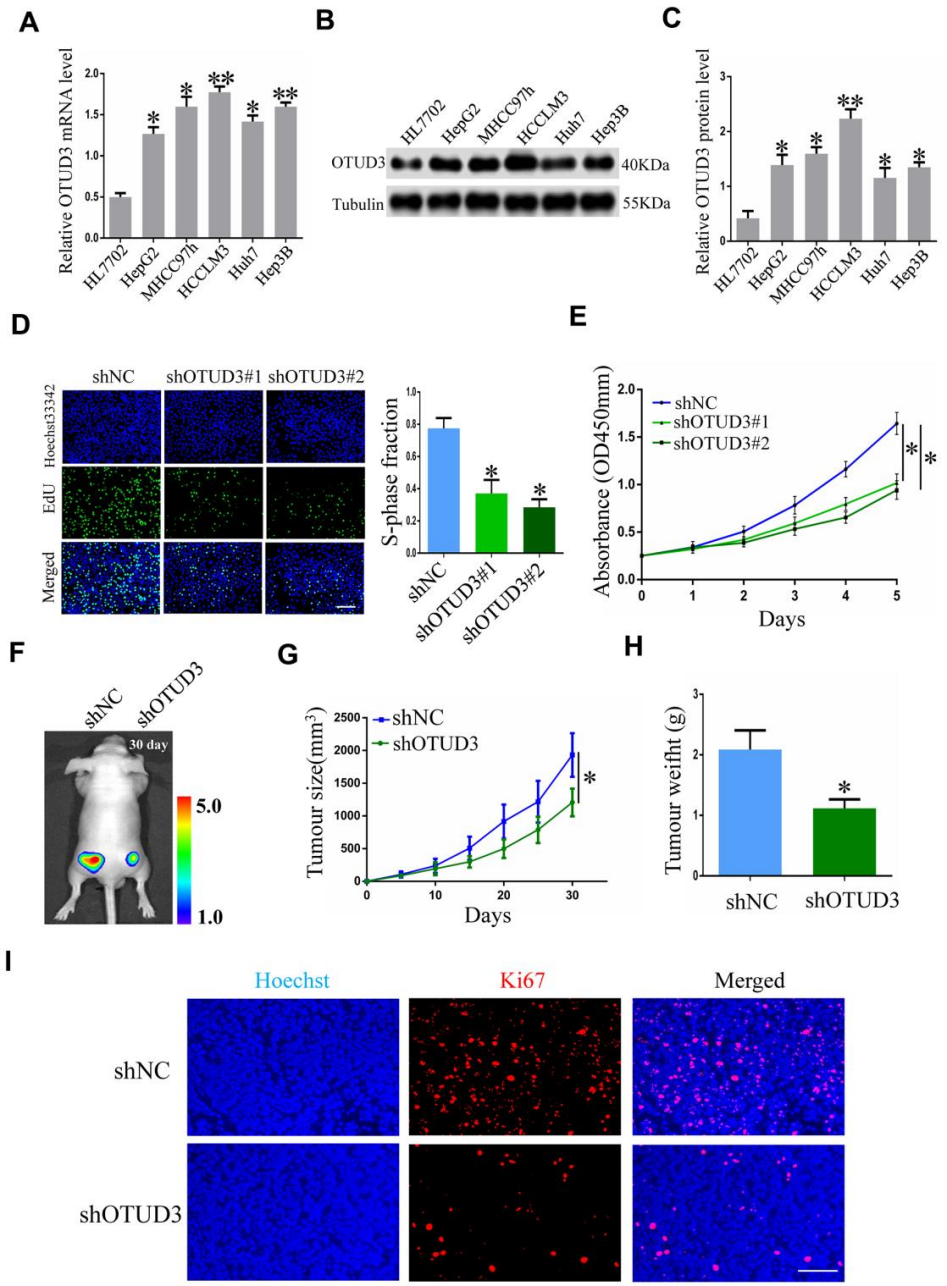
**OTUD3 knockdown suppresses HCC cell growth *in vitro* and *in vivo*.** (**A**) qRT-PCR analysis of OTUD3 mRNA level in normal liver cell HL7702 and HCC cell lines. (**B**, **C**) Western blot showing the protein expression of OTUD3 in normal liver cell HL7702 and HCC cell lines. (**D**) EdU assay evaluating the proliferation ability for HCCLM3 cells transfected with control shRNA or shRNA targeting OTUD3. Right panel is quantification of the results of the EdU assay. (**E**) CCK8 examining the effect of OTUD3 knockdown on the proliferation of HCCLM3 cell. (**F**) Luciferase intensity of the nude mice (n=6 per group) injected with luciferase-expressing HCCLM3 cells stably transfected with shNC or shRNA targeting OTUD3 were detected by IVIS, Representative images obtained are shown. (**G**, **H**) Tumour sizes and tumour weights of HCCLM3-shNC or HCCLM3-shOTUD3 group of nude mice were measured and corresponding tumour growth curves were obtained. (**I**) Immunofluorescence staining of Ki67 in subcutaneously tumour of nude mice injected with HCCLM3 cells stably transfected shOTUD3 or shNC (Scale bar: 100μm; Magnification: 200X). *P< 0.05, **P<0.01 by t-tests.

To obtain insight into the function of OTUD3 in facilitating HCC cell proliferation *in vitro*, we explored the impact of OTUD3 upregulation and downregulation on tumor cell proliferation. Initially, we examined OTUD3 expression in normal live HL7702 cells and HCC cell lines and compared the expression of OTUD3 in different cell lines. Our data indicated that OTUD3 mRNA and protein expression was dramatically upregulated in HCC cell lines (Figure 2A–2C). Additionally, the knockdown efficiency was verified through western blot and qRT-PCR (Supplementary Figure 1A, 1B). Through EdU proliferation assay and CCK-8 assay, we observed that stable OTUD3 interference effectively suppressed HCCLM3 and HepG2 cells proliferation (Figure 2D, 2E, Supplementary Figure 2A, 2B). In contrast, the EdU and CCK-8 assays showed that OTUD3 overexpression notably enhanced the proliferation of Huh7 cells (Supplementary Figure 3A, 3B).

To further determine the biological function of OTUD3 in HCC cell proliferation, we used a xenograft mouse model to assess the role of OTUD3 in HCC growth. Nude mice were injected subcutaneously with luciferase-labeled control or OTUD3 stable knockdown HCCLM3 cells to enable monitoring of HCC growth. Imaging analysis by IVIS indicated that the tumor size of mice in the shOTUD3 group was much smaller compared with the control group on the 30th day (Figure 2F). Furthermore, our data showed that stable OTUD3 interference in HCCLM3 cells contributed to a decreased tumor volume and weight (Figure 2G, 2H). Additionally, we performed immunofluorescence staining to detect the expression of the cell proliferation biomarker Ki67 using subcutaneous xenograft tissue sections from nude mice. Our results demonstrated that Ki67 expression was markedly lower in the shOTUD3 group compared with the control group (Figure 2I). Moreover, Huh7 cells with stable OTUD3 overexpression and control Huh7 cells were inoculated into nude mice subcutaneously. Our data demonstrated that the tumors of nude mice in the OTUD3 overexpression group grew much quicker and to higher weights compared with the control group (Supplementary Figure 4A, 4B). Collectively, our data reveal the crucial function of OTUD3 in controlling cell growth in HCC cells, which could inhibit HCC growth with OTUD3 knockdown.

### OTUD3 drives HCC cell invasion and migration *in vitro* and *in vivo*

**Figure 3:**
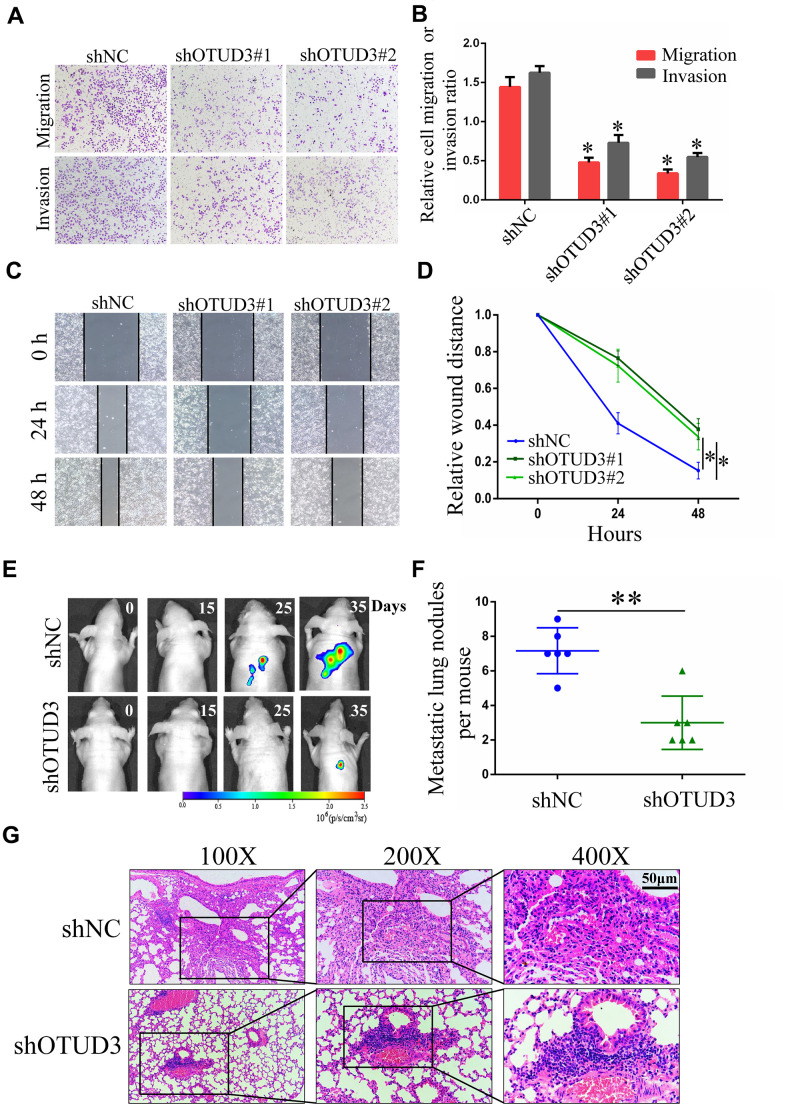
**OTUD3 downregulation inhibits HCC cells migration and invasion *in vitro* and *in vivo*.** (**A**, **B**) Invasion and migration assays were employed to evaluate the effect of OTUD3 knockdown on HCCLM3 cells metastatic ability (Magnification 200X). (**C**, **D**) Scratching assay was performed to detect migration ability of OTUD3 knockdown HCCLM3 cells compared with the control group. (**E**) *In vivo* tumour metastasis was examined using the nude mice (n=6 per group) injected with luciferase-expressing HCCLM3 cells stably transfected with shNC or shRNA targeting OTUD3 and was detected by IVIS from day 0 to day 35. Representative images obtained are shown. (**F**) Quantification of metastatic lung nodules with shNC and shOTUD3 HCCLM3 cells by tail-vein injection. (**G**) Images of H&E staining of paraffin-embedded lung tissues from shNC and shOTUD3 nude mice group (Magnification: 100X, 200X, 400X). The unpaired two-sided Student’s t test was used for comparing between two groups of equal variances. Error bars represent mean ± SD from three independent experiments. *P<0.05, **P< 0.01.

To verify the impact of OTUD3 on HCC cell invasion and migration, we performed transwell migration and invasion assays and wound healing assays. Through the transwell migration and invasion assays, we found that stable OTUD3 knockdown significantly inhibited HCCLM3 and HepG2 cells migration and invasion (Figure 3A, 3B and Supplementary Figure 2C, 2D). Moreover, the wound healing assay also indicated that OTUD3 knockdown abated HCC cell migration capability (Figure 3C, 3D and Supplementary Figure 2E, 2F). Conversely, we found that OTUD3 upregulation markedly promoted cell metastatic ability through transwell migration and invasion assays and wound-healing assays (Supplementary Figure 3C–3F).

Next, luciferase-labeled shNC-HCCLM3 or shOTUD3-HCCLM3 cells were inoculated into the tail veins of mice. By employing IVIS to monitor and visualize progression of tumor in mice, we observed that stable OTUD3 interference markedly inhibited lung metastasis (Figure 3E). Further, the H&E-staining of serial lung sections in the shOTUD3 group had fewer number of metastatic nodules compared with the negative control group (Figure 3F, 3G). Furthermore, we injected OTUD3-overexpressing or control Huh7 cells into the caudal veins of mice. The H&E staining results showed that OTUD3 overexpression led to an increased number of lung metastatic nodules (Supplementary Figure 4C, 4D). Thus, our results reveal the oncogenic function of OTUD3 in promoting HCC cell metastasis.

### OTUD3 is correlated with ACTN4 protein expression in HCC

**Figure 4:**
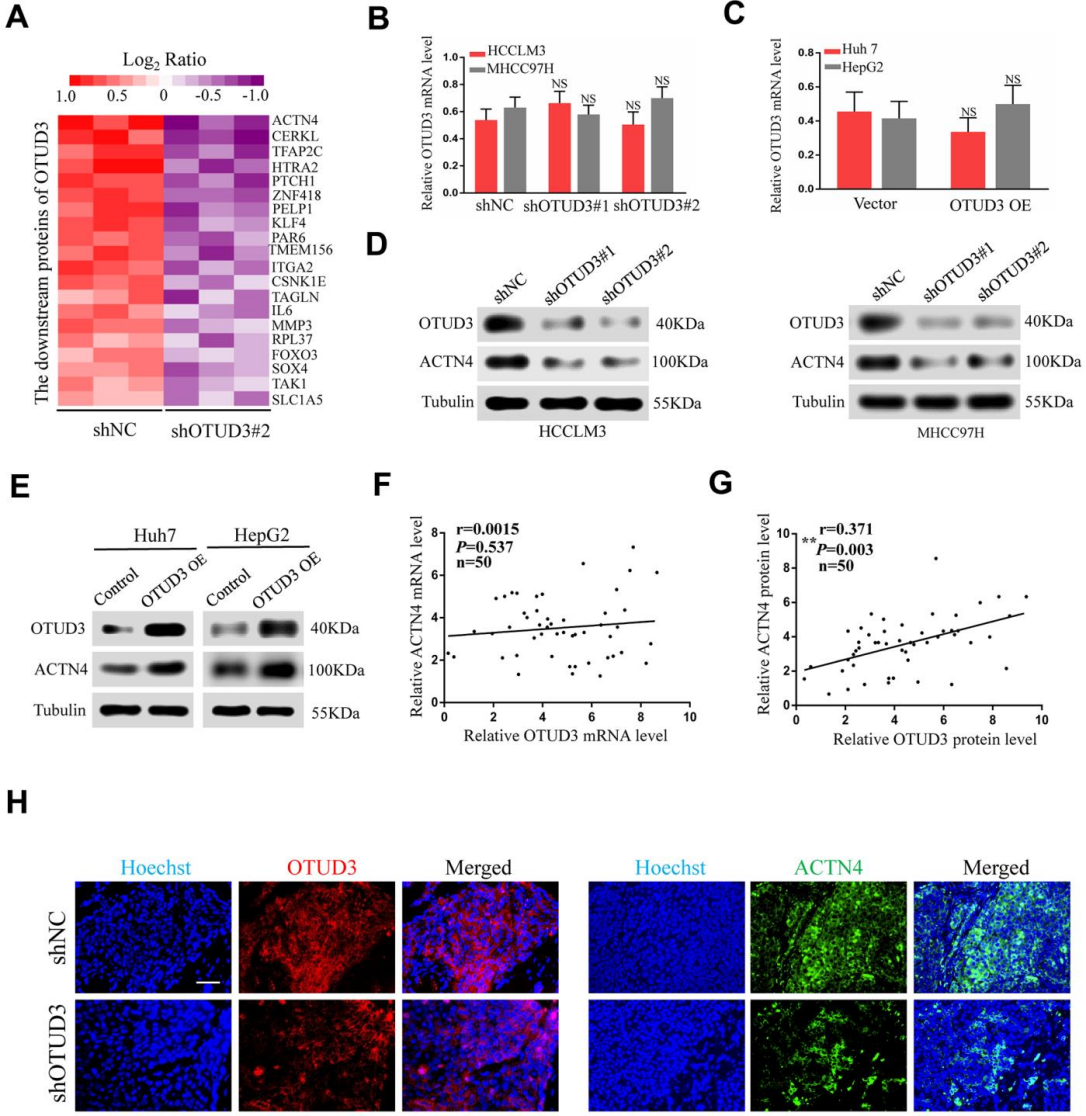
**ACTN4 protein level is significantly correlated with OTUD3 expression in HCC.** (**A**) Mass spectroscopic analysis listed the top 20 downregulated proteins with OTUD3 knockdown in HCCLM3 cells. (**B**) qRT-PCR analyses were used to detect ACTN4 mRNA levels in OTUD3 knockdown HCCLM3 and MHCC97H cells compared with the control group. (**C**) qRT-PCR analysis evaluating ACTN4 mRNA levels in OTUD3 upregulation Huh7 and HepG2 cells compared with the control group. (**D**) Western blot detecting ACTN4 protein expression in OTUD3 knockdown and the control HCCLM3 and MHCC97H cells. (**E**) Western blot showing ACTN4 protein expression in OTUD3 overexpression and the control Huh7 and HepG2 cells. (**F**) Scatter plots showed no significant correlations between OTUD3 and ACTN4 at the mRNA levels in 50 HCC tissues. (**G**) Scatter plots showed positive correlations between OTUD3 and ACTN4 at the protein levels in 50 HCC tissues. (**H**) Immunofluorescence staining of ACTN4 and OTUD3 in subcutaneously tumour of nude mice injected with HCCLM3 cells stably transfected shOTUD3 or shNC (n=6 per group; scale bar: 100μm; Magnification: 200X). The unpaired two-sided Student’s t test was used for comparing between two groups of equal variances. Error bars represent mean ± SD from three independent experiments. NS: not significant, **P< 0.01.

To better characterize the molecular mechanism by which OTUD3 regulates HCC cell progression, we analyzed tandem mass tag (TMT)-mass spectrometry proteomics data to determine the expression patterns of proteins affected by OTUD3 downregulation. Our data showed that the ACTN4 protein level was dramatically downregulated (Figure 4A). To confirm our findings, we further determined the relationship between OTUD3 and ACTN4 mRNA and protein expression in HCC cells. Interestingly, the qRT-PCR results showed that neither OTUD3 downregulation nor upregulation had a significant effect on ACTN4 mRNA levels in HCC cells (Figure 4B, 4C). In comparison, the western blot results demonstrated that OTUD3 downregulation could decrease the protein level of ACTN4, whereas OTUD3 overexpression had the opposite effect in HCC cells (Figure 4D, 4E). Furthermore, we examined the correlation between OTUD3 and ACTN4 expression in HCC tissues. As expected, the scatter plot analysis showed no significant correlation between OTUD3 and ACTN4 mRNA levels, whereas a positive relationship between OTUD3 and ACTN4 protein levels was observed (Figure 4F, 4G). Moreover, we performed immunofluorescence staining to detect OTUD3 and ACTN4 expression in mouse serial xenograft tissue sections. Our results showed that OTUD3 and ACTN4 were downregulated simultaneously in the shOTUD3 group in comparation with the control group (Figure 4H).

### OTUD3 deubiquitinates ACTN4 and maintains stabilization of ACTN4

**Figure 5:**
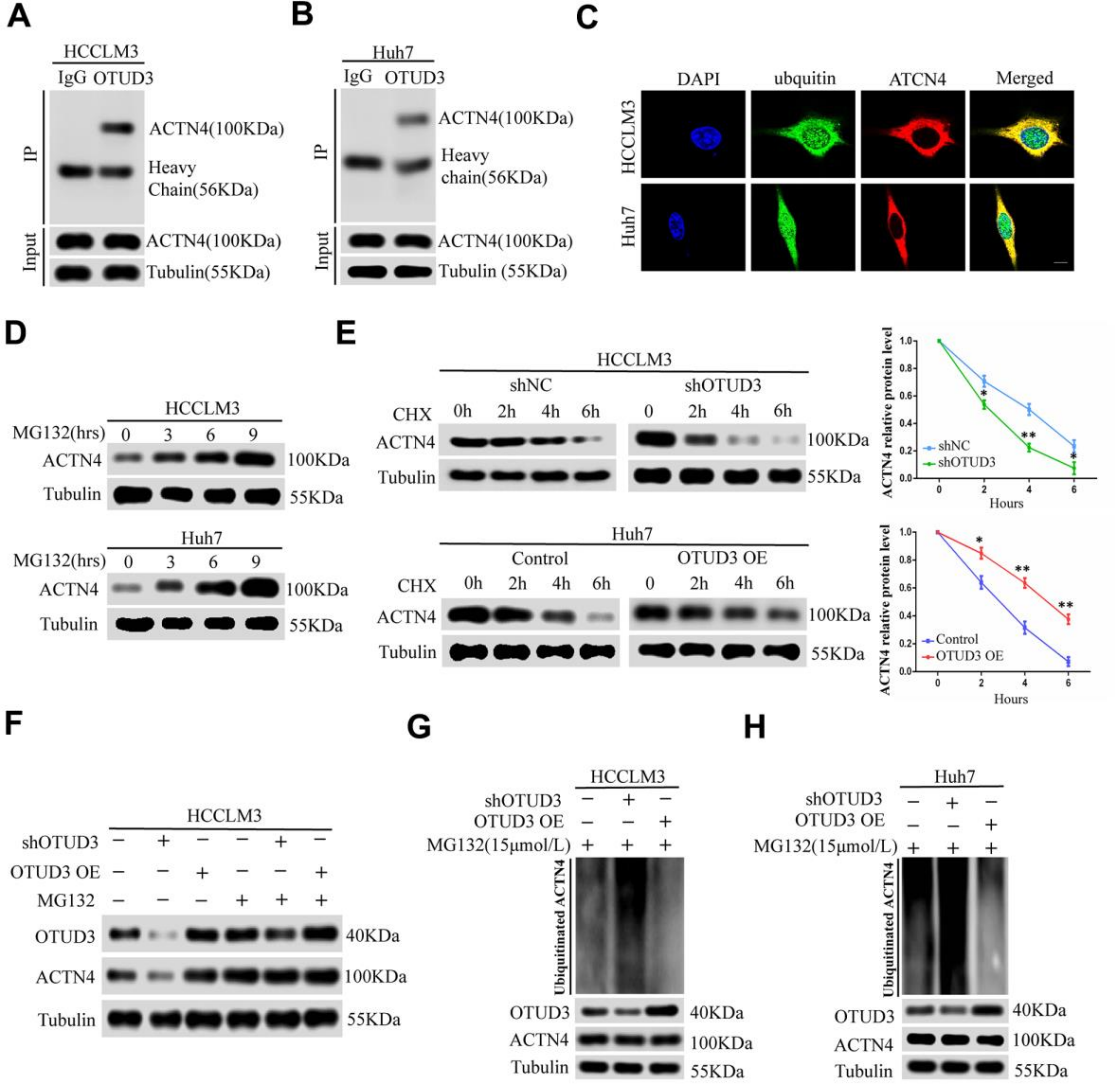
**OTUD3 enhances the stability of ACTN4 through deubiquitylation.** (**A**, **B**) co-IP experiments between endogenous OTUD3 and ACTN4 in HCCLM3 and Huh7 cells. ACTN4 was detected in the immunoprecipitation when the anti-OTUD3 antibody was used as bait. (**C**) Colocalization of OTUD3 and ACTN4 in HCCLM3 and Huh7 cells (Scale bar: 14μm). (**D**) HCCLM3 and Huh7 cells were treated with 15μM proteasomal inhibitor MG132 for the indicated time, and the levels of ACTN4 were then detected. (**E**) HCCLM3 cell transfected with OTUD3 shRNA or shNC together with stably OTUD3 overexpressing Huh7 cells and negative control were treated with 20μM cycloheximide (CHX). Cells were collected at different time points and were detected ACTN4 protein expression. (**F**) HCCLM3 cells with OTUD3 knockdown or OTUD3 overexpression were treated with MG132 (15μM). Cells were collected at 6 h and immunoblotted with the antibodies indicated. (**G**, **H**) the knockdown or upregulation of OTUD3 altered the ubiquitination of ACTN4 in both HCCLM3 and Huh7 cells. The cells in each group were treated with MG132 (15μM). The levels of ubiquitin-attached ACTN4 were detected by western blot analysis with ubiquitin (Ub) antibody.

Having confirmed the correlation between OTUD3 and ACTN4, we aimed to explore their interaction. ACTN4 has been shown to be degraded through the ubiquitin-proteasome pathway and can be stabilized in human glioblastoma [12]. Given the role of OTUD3 as a deubiquitinase and its function in stabilizing GRP78 through deubiquitylation in lung cancer cells [10], we hypothesized that OTUD3 might deubiquitinate ACTN4 and stabilize it. As expected, co-IP experiments indicated that OTUD3 could bind ACTN4 directly in HCCLM3 and Huh7 cells (Figure 5A, 5B). In addition, we performed co-IP experiments to confirm the interaction between ACTN4 and ubiquitin in HCC cells. Our data showed that ACTN4 could bind ubiquitin directly (Supplementary Figure 5A, 5B). Consistently, confocal microscopy analysis confirmed the colocalization of ACTN4 and ubiquitin in HCCLM3 and Huh7 cells (Figure 5C). We further demonstrated that ACTN4 could be degraded through the ubiquitin-proteasome pathway by using the proteasome inhibitor MG132 (Figure 5D).

To determine whether OTUD3 can block ACTN4 degradation through the ubiquitin-proteasome pathway, we treated HCCLM3 cells with OTUD3 stable knockdown and OTUD3-overexpressing Huh7 cells with a 20 μM dose of the translation inhibitor cycloheximide (CHX). At the indicated time, we detected the ACTN4 protein level and found that OTUD3 downregulation increased the ACTN4 degradation rate, whereas OTUD3 upregulation showed the opposite effect, compared with the negative control (Figure 5E). Our results indicated that neither knockdown nor upregulation of OTUD3 had a significant effect on the ACTN4 protein level in HCCLM3 cells treated with MG132 compared with those not treated with MG132 (Figure 5F). Finally, our data revealed that OTUD3 inhibition dramatically upregulated the ubiquitination level of ACTN4, while OTUD3 overexpression showed the opposite impact on ACTN4 (Figure 5G, 5H). Thus, our findings demonstrate that OTUD3 can act as a deubiquitinase of ACTN4 and stabilize it.

### ACTN4 is indispensable for HCC cell progression and functions in an OTUD3-dependent manner

To address the potential role of ACTN4 in OTUD3-mediated HCC carcinogenesis, we performed rescue experiments and investigated whether ACTN4 is a critical downstream target of OTUD3 in HCC cells. Through western blot, we found that OTUD3 downregulation dramatically abated the increased protein level of ACTN4 in HCCLM3 cells (Figure 6A). Furthermore, an EdU proliferation assay showed that OTUD3 inhibition markedly suppressed the enhanced HCC cell growth induced by ACTN4 upregulation (Figure 6B, 6C). Simultaneously, transwell migration and invasion assays demonstrated that ACTN4 upregulation enhanced HCC cell metastatic ability, whereas OTUD3 knockdown reversed this trend effectively (Figure 6D). To further confirm our findings, we transfected ACTN4-silencing plasmids into OTUD3-overexpressing HCC cells and found that the decreased protein level of ACTN4 was rescued by OTUD3 overexpression (Figure 6E). Consistently, EdU assay and transwell migration and invasion assays indicated that the suppression of HCC cell growth and metastatic capabilities induced by ACTN4 interference was reversed by OTUD3 upregulation (Figure 6F–6H). These findings reveal that ACTN4 is crucial for OTUD3-driven HCC cell progression *in vitro*.

**Figure 6:**
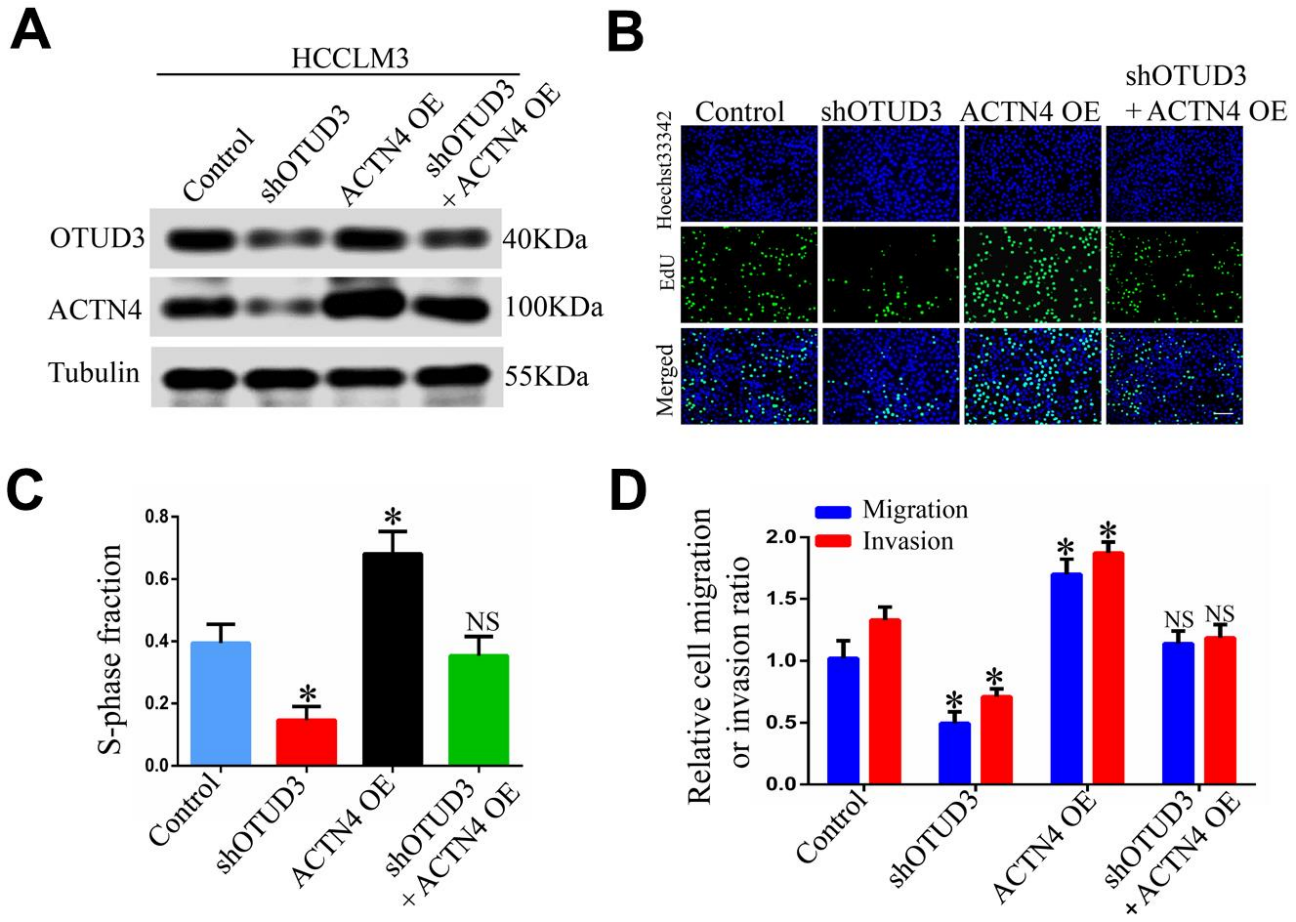
**ACTN4 is critical for OTUD3-mediated HCC cells progression *in vitro* and *in vivo*.** (**A**) Western blot confirming the downregulation of OTUD3 abated increased ACTN4 expression in HCCLM3 cells. (**B**, **C**) EdU assay evaluating the effect of OTUD3 knockdown on accelerated HCC cell growth enhanced by ACTN4 upregulation. (**D**) Quantification of HCC cell transwell migration and invasion results.

**Figure 6:**
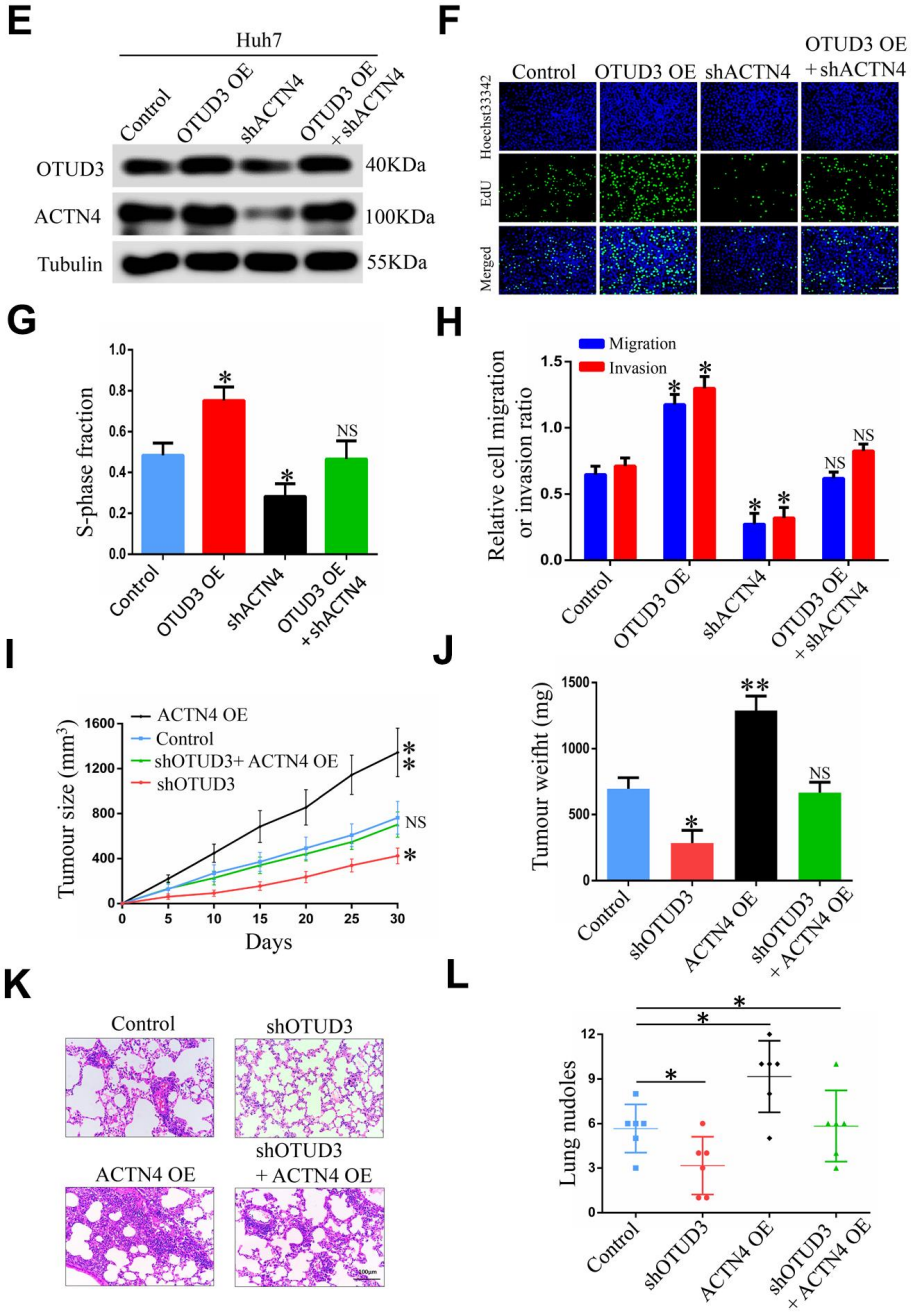
**ACTN4 is critical for OTUD3-mediated HCC cells progression *in vitro* and *in vivo*.** (**E**) Western blot showing the overexpression of OTUD3 rescued the decreased ACTN4 protein level caused by ACTN4 knockdown. (**F**, **G**) EdU assay identifying the effect of OTUD3 upregulation on inhibited HCC cell growth caused by ACTN4 downregulation. (**H**) Quantification of HCC cell transwell migration and invasion results. (**I**, **J**) Tumor sizes and tumor weights of 24 nude mice (6 mice per group) were measured and corresponding tumor growth curves were obtained in the rescue experiments. (**K**, **L**) *In vivo* lung metastasis rescue experiment was examined in 24 nude mice (6 mice per group). Representative H&E staining of lungs are shown (Scale bar: 100μm; magnification: 200X), along with the number of lung metastases in the four groups of nude mice. The unpaired two-sided Student’s t test was used for comparing between two groups of equal variances. Error bars represent mean ± SD from three independent experiments. NS: not significant, *P< 0.05, **P< 0.01.

To further verify whether ACTN4 played the same role *in vivo*, we constructed tumorigenicity and tail vein injection mouse models. Strikingly, we found that ACTN4 upregulation significantly promoted tumor growth, while OTUD3 knockdown effectively inhibited this trend (Figure 6I, 6J). Similarly, HCCLM3 cells with stable OTUD3 knockdown dramatically lost the enhanced metastatic ability induced by ACTN4 overexpression (Figure 6K, 6L). Herein, our findings reveal that ACTN4 is essential for OTUD3-drived HCC cell carcinogenesis *in vivo*.

## DISCUSSION

Deubiquitinating enzymes play important roles in multiple cancers. Understanding the mechanisms about how these enzymes regulate cancer carcinogenesis is critical to develop therapeutic strategies. In this study, we carried out a comprehensive investigation of the function and mechanism of OTUD3 in regulating HCC progression.

Compared with other DUBs, such as BAP1 [13] and USP7 [14, 15], OTUD3 is rarely studied in cancer. Although OTUD3 has been identified as an important oncogenic driver in lung cancer carcinogenesis [10], our study is the first one disclosing the function of OTUD3 in HCC. We find that OTUD3 is highly expressed in HCC tissues and that its expression is markedly correlated with tumor size, distant metastasis and TNM stage of HCC patients. Importantly, we disclose for the first time that ACTN4 can be stabilized by OTUD3 and thus drives HCC carcinogenesis.

Alpha-actinin 4 (ACTN4), known as an actinin-binding protein, belonging to the spectrin superfamily, is important for the regulation of cytoskeletal integrity and cell movement [16, 17]. The novel role of ACTN4 in promoting cell motility and cancer invasion was first reported by Honda K et al. [18]. In recent years, accumulating evidence has shown that ACTN4 enhances migration and lymph node metastasis in colorectal cancer and promotes epithelial-to-mesenchymal transition and carcinogenesis of cervical cancer [19, 20]. Additionally, deubiquitinating enzyme OTUD3 belongs to OTU family and has the function of blocking protein degradation through the ubiquitin-proteosome pathway [21]. In our study, we have identified ACTN4 as an essential downstream target of OTUD3 through performing mass spectroscopic analysis. Interestingly, we also found that OTUD3 expression had a significant impact on ACTN4 expression only at the protein level in HCC cells. Although we have shown that high OTUD3 expression is correlated with poor prognosis for HCC patients, the effects of the combination of OTUD3 and ACTN4 expression on prognosis in real-world cohorts have not been analyzed. Consistently, in HCC tissues, there was a positive correlation between OTUD3 and ACTN4 at protein level whereas no correlation between OTUD3 and ACTN4 expression at mRNA level. We thus speculated that OTUD3 might have direct impact on post-translational modification of ACTN4 protein. In addition, ACTN4 can be degraded through the ubiquitin-proteosome pathway and be stabilized in human glioblastoma cells [12]. Considering the deubiquitylation function of OTUD3, we hypothesized that OTUD3 might interact with ACTN4 to deubiquitinate it. As expected, we suggested that ACTN4 could interact with OTUD3 and it could also be degraded through the ubiquitin-proteosome pathway in HCC cells. Furthermore, our data indicated that OTUD3 upregulation inhibited ACTN4 ubiquitination whereas OTUD3 knockdown showed the opposite effect. In other words, OTUD3 can deubiquitinate ACTN4 to inhibit its degradation in HCC cells.

Moreover, our study has also revealed that OTUD3 drives HCC proliferation and metastasis in an ACTN4-dependent manner. In prostate cancer, ACTN4 was found to be essential for WAVE2 (WASP family Verprolin-homologous protein)-mediated cancer cells motility and invasiveness [22]. In this study, we provided further evidence to demonstrate that ACTN4 was critical for HCC cells proliferation and metastasis driven by OTUD3 through rescue experiments. Through upregulating ACTN4 expression, decreased growth and metastatic ability of HCC cells induced by OTUD3 knockdown was rescued whereas downregulating ACTN4 expression prominently inhibited OTUD3-enhanced HCC proliferation and metastasis. Furthermore, we demonstrated ACTN4 was critical for OTUD3-drived HCC progression *in vivo*.

In conclusion, our findings indicate that OTUD3 is aberrantly increased in HCC tissues and is markedly associated with worse prognosis of HCC patients. OTUD3 plays an essential role in accelerating HCC cells growth, migration and invasion *in vitro* and *in vivo*. Mechanistically, OTUD3 deubiquitinates and stabilizes ACTN4 in HCC cells. In addition, ACTN4 is key for OTUD3-mediated HCC cells progression. This study contributes to our ever-increasing understanding of the function of OTUD3 in malignant carcinoma and highlight the potential role of OTUD3 as a prognostic indicator and a therapeutic target in HCC.

## MATERIALS AND METHODS

### Patients and human specimens

Paraffin-embedded and fresh human HCC specimens were from 115 patients undergoing HCC resection at the Jiangxi Province Tumor Hospital of Nanchang University from June 2013 to July 2020. Liquid nitrogen was employed to freeze fresh specimens and all of the samples were stored at -80° C for research. Research ethics committee of the Jiangxi Province Tumor Hospital of Nanchang University has permitted the research and informed consent of the patients was obtained. All patients were followed up for 5 years.

### Cell culture and treatment

HCC cell lines including Huh7, MHCC97H, HepG2, HCCLM3, Hep3B and human normal hepatocyte cell lines HL-7702 were obtained from Cell Bank of Type Culture Collection of Chinese Academy of Sciences and the Shanghai Institute of Cell Biology in China. The identity of the cell lines was confirmed by short tandem repeat analysis. All cell lines were cultured in Dulbecco’s modified Eagle’s Medium (Gibco) containing 10% fetal calf serum (FBS, HyClone, USA) at 37° C in a humidified incubator containing 5% CO2.

### Quantitative real-time PCR (qRT-PCR)

Total RNA was extracted by the standard Trizol-based protocol (Invitrogen, USA). Complementary DNA (cDNA) was synthesized using the PrimeScript RT Reagent Kit (Invitrogen, USA) and SYBR Premix Ex Taq (TaKaRa Bio, Shiga, Japan) was employed in qRT-PCR, according to the manufacturer's instructions. Supplementary Table 1 has shown the information of gene-specific primers.

### Western blot

Western blot was performed as previous study [15]. Extraction of total cellular proteins was extracted by RIPA buffer (Beyotime, Shanghai, China) containing protease and inhibitor mixes (Thermo Fisher Scientific, New York, USA) on ice. BCA Protein Assay kit (Thermo Scientific, Waltham, MA, USA) was performed to evaluate protein concentration. Equal amounts of proteins were separated by sodium dodecylsulfonate (SDS) polyacrylamide gel electrophoresis and transferred onto a polyvinylidene fluoride (PVDF) membrane by electroblotting (Millipore, Bedford, MA, USA). Primary antibodies were added and incubated throughout a night at 4° C. Primary antibodies including anti-OTUD3 monoclonal antibody (1:500; HPA028543, Sigma), anti-ACTN4 monoclonal antibody (1:1000, 15145, CST). After being incubated with the second antibody (CST, MA, USA) for 1h at room temperature, the intensity of protein bands was evaluated by Quantity One software (Bio-Rad, Hercules, CA, USA).

### Immunohistochemistry (IHC) staining

Paraffin-embedded sections (4 mm thick) of human HCC tissues and normal adjacent tissues were deparaffinized at 70° C for 25mins. Next, sections were subjected to antigen retrieval in microwave-heated citrate buffer (pH6.0) for 30 mins. After incubation for 30 mins in goat serum (Solarbio, Beijing, China), tissued sections were incubated by primary antibodies overnight at 4° C. Next, HRP-conjugated secondary antibody (Boster) was used to incubate sections for 2h at room temperature. At last, DAB Detection Kit (Maxim) was adopted for immunostaining for 2 mins. The proportion of positive areas were scored semi-quantitatively by 3 pathologists who were blind to the clinical parameters. In brief, 100 cells were counted randomly at 200X microscopic fields and were classified into five groups according to the percentage of positive staining cells in HCC tissues as follows: 0 = negative; 1 – 3 = 1 – 25%; 4 – 6 = 26 – 50%; 7 – 9 = 51 – 75%; 10 – 12 = ≥76%. The score ranging from 0 to 6 was considered as a low-expression group, whereas the score ranging from 7 to 12 was considered as a high-expression group. Additionally, the prognostic value of OTUD3 expression is also defined through the immunohistochemistry score.

### Stable cell lines and plasmids

HCC cell lines with stable OTUD3 overexpression (OE) or knockdown were established by transfection of lentivirus containing OTUD3 overexpression plasmid (GV640 vector, Genechem, Shanghai, China) or short hairpin RNA (shRNA) (pGLVH1 vector, GenePharma, Shanghai, China). Cells infected by lentivirus were selected using puromycin (Invitrogen, Carlsbad, CA, USA) for one month. Transient OTUD3 overexpression or knockdown was performed by transfections of OTUD3 OE or knockdown plasmids using Lipofectamine 2000 Transfection Reagent (Invitrogen, Carlsbad, CA, USA) following the manufacturer's recommended protocol [23].

### Immunofluorescence staining

Paraffin-embedded sections of xenografted tumour tissues were deparaffinized, followed by antigen retrieval in microwave-heated EDTA buffer (pH8.0) for 30 mins. After incubation for 15 mins in 0.1% TritonX-100 (Solarbio, Beijing, China) and 1% goat serum (Solarbio, Beijing, China) for 30 mins, tissued sections were incubated in dark by primary antibodies overnight at 4° C, followed by secondary incubation for 1h with Alexa Fluor 594 goat anti-rabbit IgG (1:500, Life Technologies). Nuclear were stained with Hoechst 33342 (Life Technologies) for 1 min. Tissues were observe through a confocal laser scanning microscope (SP-II; Leica Microsystems, Wetzlar, Germany).

### EdU assay

Cells were seeded in 96-well plates at an initial concentration of 5x10^4^ cells per well. After culturing for 24 hours, 5-ethynyl-20-deoxyuridine (EdU; Ribobio) was used to culture cells for 2 hours. After cells being incubated with 1xApollo reaction cocktail for 30 minutes, Hoechst 33342 (5 mg/mL) was used to stain the DNA contents of the cells in each well for 25 minutes and was visualized through a confocal laser scanning microscope (SP-II; Leica Microsystems, Wetzlar, Germany).

### Cell counting kit-8 assay

CCK8 assay was employed to evaluate cell viability after 24, 48, 72, 96, 120h. Transfected cells were seeded into 96-well plates at an initial concentration of 3x10^3^ cells per well. According to the manufacturer’s instructions, 10μl of CCK-8 solutions (Dojindo Laboratories, Kumamoto, Japan) was added to each well. After incubation in cell incubator for 1h, the absorbance at wavelength of 450 nm was recorded.

### The wound-healing assay

Transfected cells were incubated into 6-well plates until growing to 80% to 90% confluence. Then 200μl pipette tip was used to scratch across the cells surface followed by three washed with PBS. Subsequently, the cells were incubated at 37° C and the wound range was imaged by phase-contrast photography at 0h, 24h and 48h. Three randomly selected wound areas were analyzed.

### *In vitro* migration and invasion assays

Cell migration assay and invasion assays were performed through a transwell system (Corning, NY, USA) with or without Matrigel matrix (BD bioscience) coated above the membrane. Stably transfected cells were suspended in pure DMEM at a concentration of 1x10^5^ /ml. 500μl cell suspension was added in the upper chamber. Fresh medium containing 10% FBS was added in the lower chamber as a chemoattractant. After incubation for 48h, the non-migrated cells on the upper surface of the membrane were removed, and the cells on the lower surface were fixed with methanol and stained by 0.1% crystal violet. The cells in five random microscopic fields were counted and imaged using a light microscope with a DP70 CCD system (Olympus Corp).

### Co-immunoprecipitation experiment

Cell lysis was incubated with 50μl protein A+G Agarose (Thermo Scientific) and 1 μg of the indicated antibody overnight at 4° C. The protein A/G-agarose were collected by centrifugation. After adding with loading buffer, the protein was heated for 15 mins at 100° C. Then the immunoprecipitated proteins were examined by SDS-PAGE and immunoblotting analysis. The intensity of protein bands was analyzed by Quantity One software (Bio-Rad, Hercules, CA, USA).

### Xenografts mouse model

HCCLM3 cells (2 x10^6^ cells per mouse) expressing a luciferase reporter stably transfected with LV-shNC or LV-shOTUD3 lentivirus were injected into the flanks of 8-week-old female BALB/c-nude mice (n=6 per group) subcutaneously. The *in vivo* imaging system (IVIS, PerkinElmer, USA) was employed to monitor and image the growth of tumours regularly. Tumour volume was measured every 5 days using the formula: V = [length/2] × [width^2^] [24]. Finally, all of the tumour xenografts were harvested and weighted at the 30th day. All animals were randomly divided into different groups by a technician under blinding condition. Animal experiments were approved by the Ethics Committee for Animal Experiments of the Second Affiliated Hospital of Nanchang University.

### Metastasis model

HCCLM3 cells (2 x10^6^ cells per mouse) expressing a luciferase reporter stably transfected with LV-shNC or LV-shOTUD3 lentivirus were injected into the tail vein of 8-week-old female BALB/c-nude mice (n=6 per group). IVIS was employed to monitor and photograph the tumour progression in mice. Organs of mice were harvested after 5 weeks and metastatic nodes in lung sections were evaluated after HE staining.

All mice were randomized into different groups by a technician under blinding condition. Animal experiments were approved by the Ethics Committee for Animal Experiments of the Second Affiliated Hospital of Nanchang University.

### Statistical analysis

All results were presented as mean ± SD. Log-rank test was employed to analyze survival of patients. Student’s t-test was employed in statistical analyses between two groups. One-way ANOVA was employed for multiple comparisons. GraphPad Prism (version 5) was used in all statistical analyses and *P*< 0.05 was considered significant.

## Supplementary Material

Supplementary Figures

Supplementary Table 1
