# Supplementary material for: The deubiquitinase OTUD3 stabilizes ACTN4 to drive growth and metastasis of hepatocellular carcinoma
Source: Aging (Albany NY). 2021 Aug 10;13(15):19317–38. doi: 10.18632/aging.203293 (PMC8386523; doi:10.18632/aging.203293)
Supplement: Supplementary Figures [file aging-13-203293-s001.pdf]

SUPPLEMENTARY FIGURES

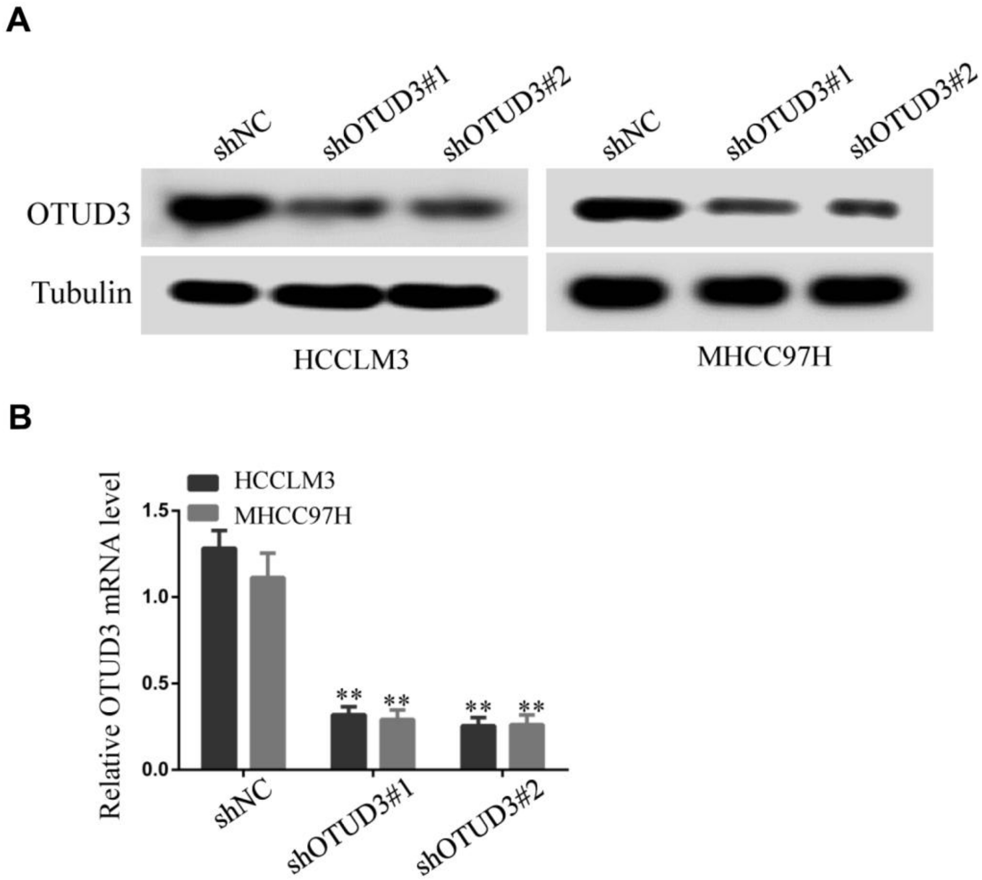

Supplementary Figure 1. OTUD3 shRNA transfection efficiency. (**A**, **B**) The OTUD3 knockdown efficiency was confirmed by western blot and qRT-PCR analysis. Data were mean ± S.D. of three independent determinations. $^{**}P < 0.01$ of t-tests.

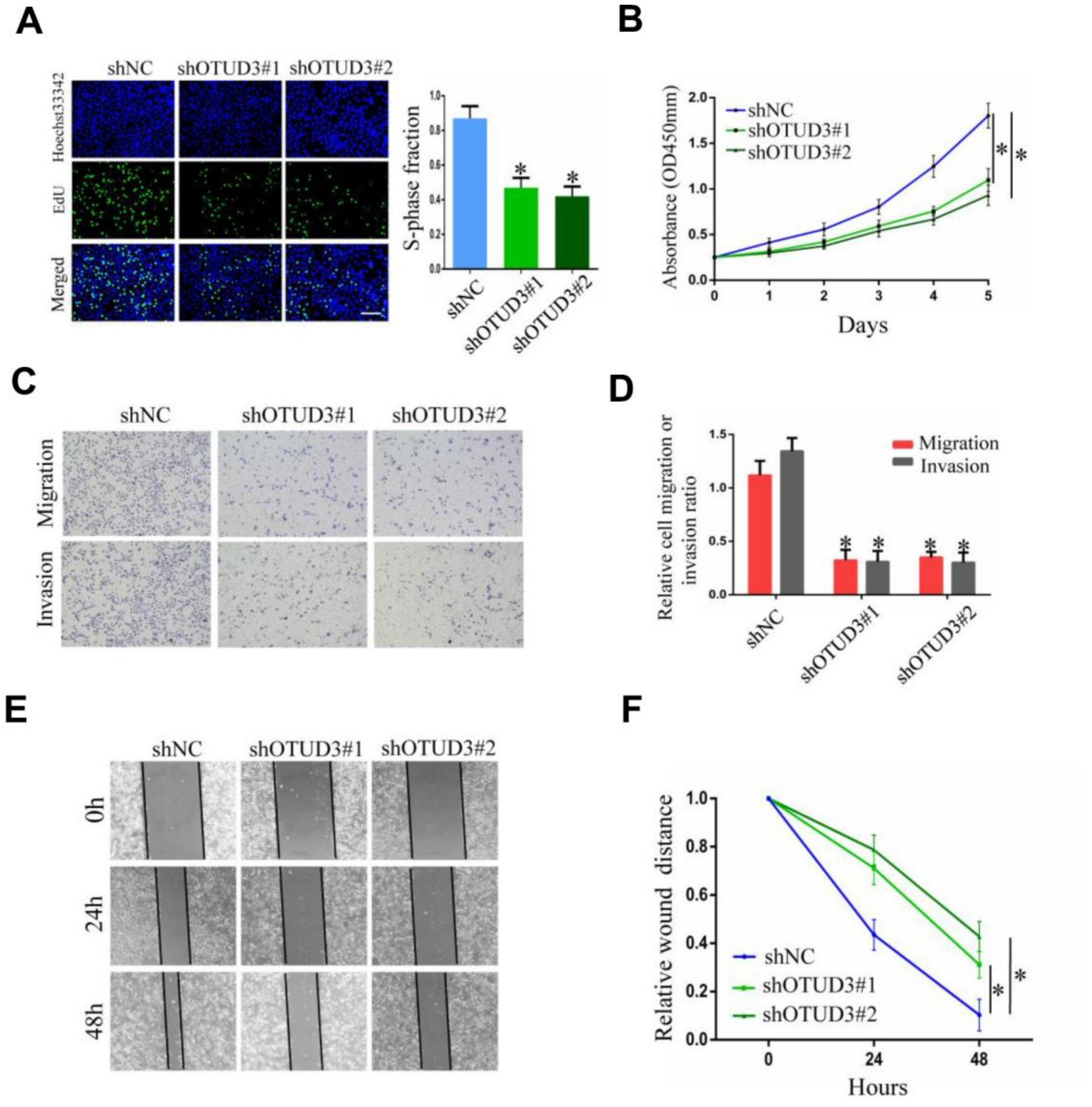

Supplementary Figure 2. OTUD3 suppression inhibits HCC cells proliferation, migration and invasion. (**A**) EdU assay evaluating the proliferation ability for HepG2 cells transfected with control shRNA or shRNA targeting OTUD3. Right panel is quantification of the results of the EdU assay. (**B**) CCK8 examining the effect of OTUD3 knockdown on the proliferation of HepG2 cell. (**C**, **D**) Invasion and migration assays were employed to evaluate the effect of OTUD3 knockdown on HepG2 cells metastatic ability (Magnification 200X). (**E**, **F**) Scratching assay was performed to detect migration ability of OTUD3 knockdown HepG2 cells compared with the control group.

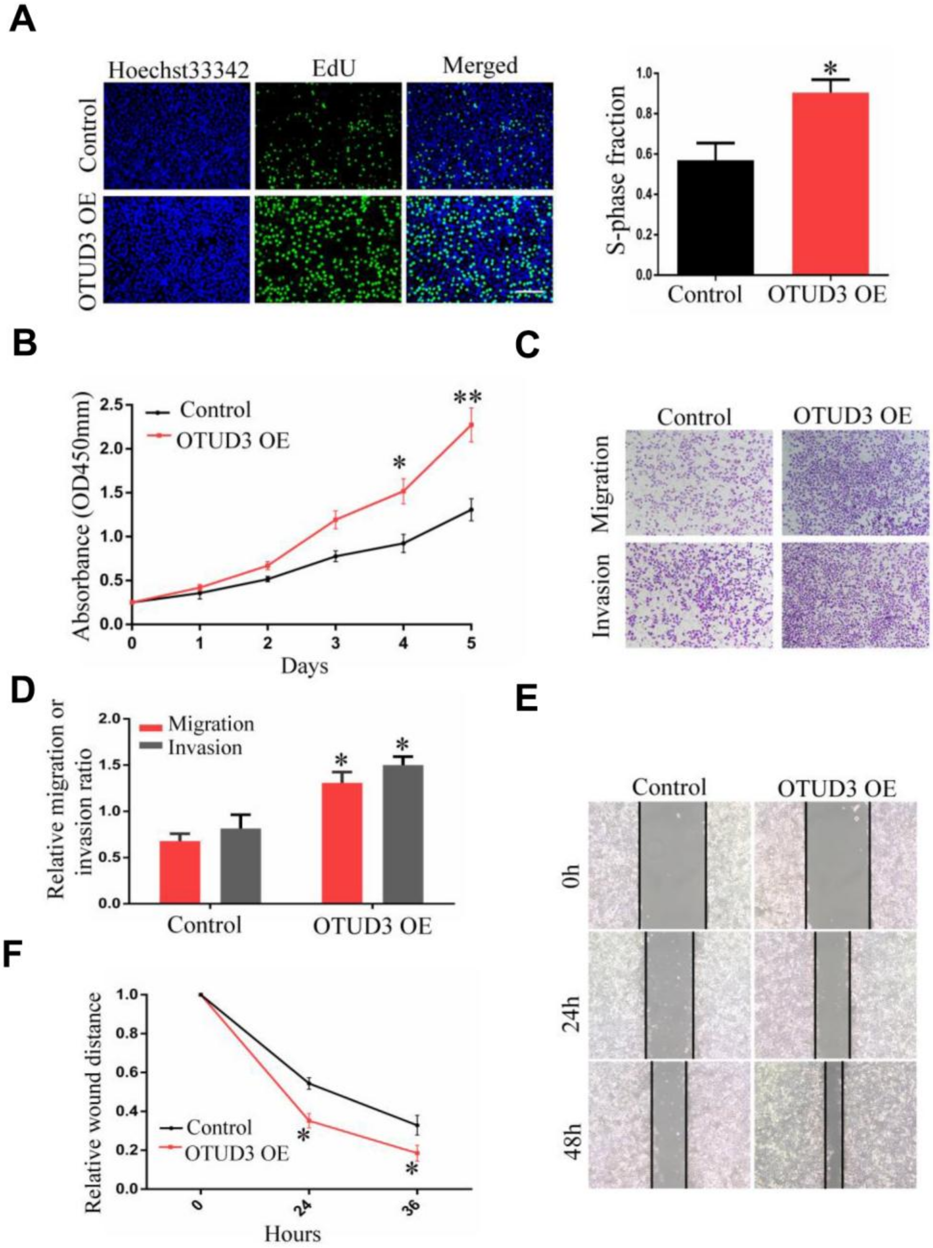

Supplementary Figure 3. OTUD3 overexpression facilitates HCC cells proliferation, migration and invasion *in vitro.* (**A**–**C**), EdU assay and CCK8 experiments showing cell proliferation capacity of OTUD3 overexpressing Huh7 cell compared with the control group. (**D**–**F**) Transwell migration and invasion assay and wound healing assay evaluating the metastatic ability of OTUD3 overexpressing Huh7 cell compared with the control group. Data were mean ± S.D. of three independent determinations. *$P < 0.05$, **$P < 0.01$ of t-tests.

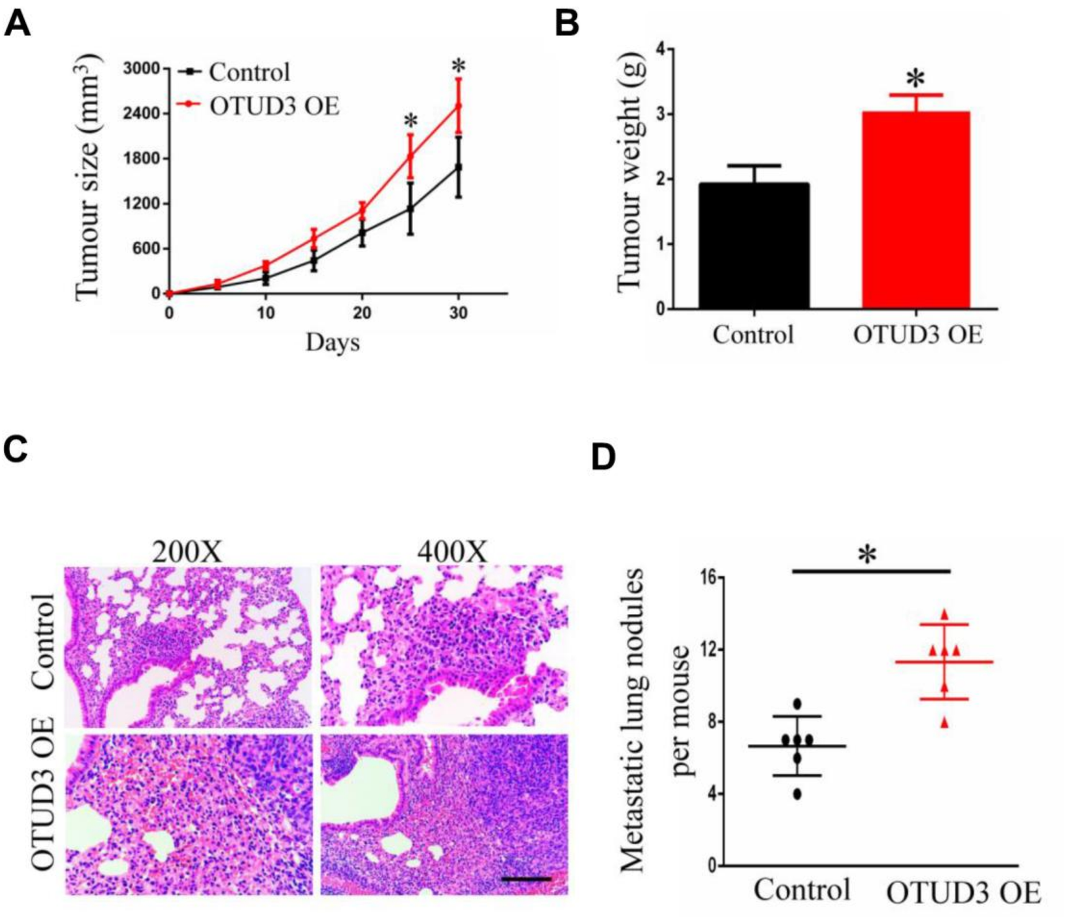

Supplementary Figure 4. OTUD3 overexpression accelerates HCC cells proliferation, migration and invasion *in vivo*. (**A**, **B**) Tumour sizes and tumour weights of the control or Huh7-OTUD3 OE groups (n=6 per group) of nude mice were measured and corresponding tumour growth curves were obtained. (**C**) Images of H&E staining of paraffin-embedded lung tissues from the control or Huh7-OTUD3 OE nude mice group (n=6 per group) (Magnification: 200X, 400X; scale bar: 50μm). (**D**) Quantification of metastatic lung nodules with the control or Huh7-OTUD3 OE cells by tail-vein injection. Data were mean ± S.D. of three independent determinations. *$P < 0.05$ of t-tests.

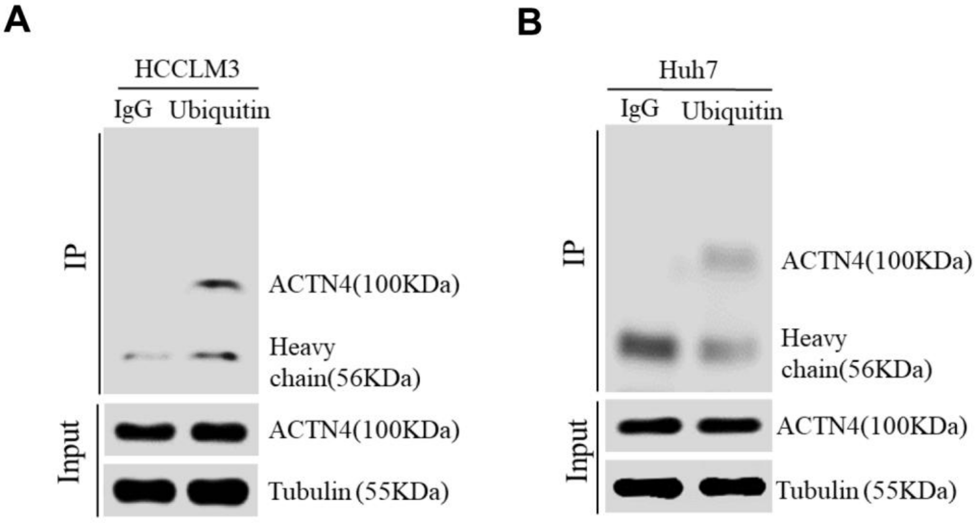

Supplementary Figure 5. ACTN4 bind ubiquitin directly in HCC cells. (**A**, **B**) co-IP experiments between endogenous ubiquitin and ACTN4 in HCCLM3 and Huh7 cells. ACTN4 was detected in the immunoprecipitation when the anti-ubiquitin antibody was used as bait.
