# Supplementary material for: The deubiquitinase OTUD3 stabilizes ACTN4 to drive growth and metastasis of hepatocellular carcinoma
Source: Aging (Albany NY). 2021 Aug 10;13(15):19317–38. doi: 10.18632/aging.203293 (PMC8386523; doi:10.18632/aging.203293)
Supplement: Supplementary Table 1 [file aging-13-203293-s002.pdf]

SUPPLEMENTARY TABLE

Supplementary Table 1. Primers sequences and OTUD3 shRNA sequences.

Name	Sequence
OTUD3 forward	5'- GGCCGCTAATACGACTCACT-3′
OTUD3 reverse	5'- TTCTGTGGCTGAAAACCCCT-3′
ACTN4 forward	5'- GGGCAGAAGAGATTGTGGAC-3′
ACTN4 reverse	5'- TTGTTCAGGTTGGTGACAGG -3′
GAPDH forward	5’-CCATGGGGAAGGTGAAGGTC-3’
GAPDH reverse	5’-TGAAGGGGTCATTGATGGCA-3’
shOTUD3-1	5'-TGGAAATCAGGGCTTAAAT-3'
shOTUD3-2	5'-GAGTTACACATCGCATATC-3'
